# Genetic-gonadal-genitals sex (3G-sex) and the misconception of brain and gender, or, why 3G-males and 3G-females have intersex brain and intersex gender

**DOI:** 10.1186/2042-6410-3-27

**Published:** 2012-12-17

**Authors:** Daphna Joel

**Affiliations:** 1 School of Psychological Sciences and Sagol School of Neuroscience, Tel-Aviv University, Jerusalem, Israel

**Keywords:** Sex differences, Gender differences, Male brain, Female brain, Intersex

## Abstract

The categorization of individuals as “male” or “female” is based on chromosome complement and gonadal and genital phenotype. This combined genetic-gonadal-genitals sex, here referred to as 3G-sex, is internally consistent in ~99% of humans (i.e., one has either the “female” form at all levels, or the “male” form at all levels). About 1% of the human population is identified as “intersex” because of either having an intermediate form at one or more levels, or having the “male” form at some levels and the “female” form at other levels. These two types of “intersex” reflect the facts, respectively, that the different levels of 3G-sex are not completely dimorphic nor perfectly consistent. Using 3G-sex as a model to understand sex differences in other domains (e.g., brain, behavior) leads to the erroneous assumption that sex differences in these other domains are also highly dimorphic and highly consistent. But parallel lines of research have led to the conclusion that sex differences in the brain and in behavior, cognition, personality, and other gender characteristics are for the most part not dimorphic and not internally consistent (i.e., having one brain/gender characteristic with the “male” form is not a reliable predictor for the form of other brain/gender characteristics). Therefore although only ~1% percent of humans are 3G-“intersex”, when it comes to brain and gender, we all have an intersex gender (i.e., an array of masculine and feminine traits) and an intersex brain (a mosaic of “male” and “female” brain characteristics).

## 

Whether a scientist or a layperson, when people think about sex differences in the brain and in behavior, cognition, personality and other gender characteristics, their model is that of genetic-gonadal-genitals sex (3G-sex, Endnote). 3G-sex is a categorization system in which ~99% of human subjects are identified as either “male” or “female”, and identification with either category entails having all the characteristics of that category (i.e., “female” = XX, ovaries, uterus, fallopian tubes, vagina, labia minora and majora, clitoris, and “male” = XY, testes, prostate, seminal vesicles, scrotum, penis). That 3G-sex is such a powerful categorization system relies on two characteristics. One is that there is an almost dimorphic division into a “male” form and a “female” form at the different levels of 3G-sex. The second is that there is a high degree of consistency between one’s form at the different levels. In other words, if one has the “female” form at the genetic level, one is highly likely to also have the “female” form at the gonadal and genitals level. Only about 1% of the human population does not fit into one of these two categories of 3G-sex (this is a conservative estimate on the basis of [1]). This can be due to either having an intermediate form at one or more levels (e.g., ovotestis, intersex external genitalia), reflecting the fact that the different levels of 3G-sex are not completely dimorphic, or having the “male” form at some levels and the “female” form at other levels (e.g., a person with “male” chromosome complement [XY], “male” gonads [testes] and “female” external genitalia, as in Complete Androgen Insensitivity Syndrome), reflecting the fact that the different levels of 3G-sex are not always consistent (Figure 1). The two types of subjects are categorized as “intersex” (e.g., [1]).

**Figure 1:**
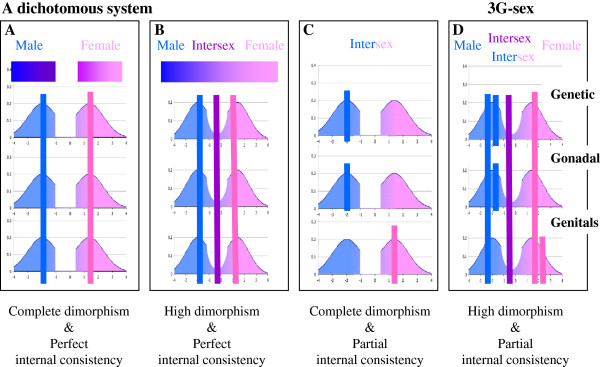
Illustrates* different combinations of complete/high dimorphism and perfect/partial internal consistency at the level of 3G-sex. A. A system with complete dimorphism (i.e., no overlap between the form in “males” and in “females”) at each level and perfect consistency between levels (i.e., one has the same type of form at all levels). The latter is represented by the pink and blue vertical bars, with each bar marking the form at each of the three levels of a single “female” and a single “male”, respectively. Only such a system would be dichotomous, that is, all subjects would be either “male” or “female”. **B.** A system with high dimorphism (i.e., some overlap between the form in “males” and in “females”) at some levels and perfect consistency between levels. In such a system there would be “male”, “female” and “intersex“ subjects. The latter will be characterized by having the “intersex” form at all levels (an example for such a subject is represented by the purple vertical bar). In such a system subjects can be aligned on a “male”-”female” continuum, as shown in the upper bar. **C.** A system with complete dimorphism at each level and partial consistency between levels. In such a system there would be “male”, “female” and “intersex” subjects, with the latter characterized by having the “male” form at some levels and the “female” form at other levels (an example for such a subject is represented by the pink and blue short bars). **D.** A system with high dimorphism at some levels and partial consistency between levels. In such a system there would be “males”, “females” and the two types of “intersex” subjects. 3G-sex is such a system. * the graphs were not created on the basis of actual distributions and are for illustration purpose only.

Using 3G-sex as a model to understand sex differences in other domains (e.g., brain, behavior) leads to the assumption that sex differences in these other domains obey the same rules, that is, are highly dimorphic and highly consistent, and that therefore belonging to a category entails having all the characteristics of that category. As a result, humans are divided into men and women and brains into male brains and female brains (e.g., [2-4]). However, this assumption does not hold true for sex differences in these other domains. In fact, it does not hold true even for sex differences in bodily characteristics beyond 3G-sex. From the level of “sex” hormones (e.g., estradiol and testosterone), through the level of secondary sex characteristics, such as breast form and facial and body hair, to the level of observable body features, such as height, a considerable overlap exists between the distribution of 3G-“females” and 3G-“males”, and the consistency between the form of the different features is not as high as the consistency between the different levels of 3G-sex. For example, about 33-50% of 3G-“males” have the “female” form of breasts (i.e., Gynecomastia, [5]) together with the “male” form of facial and body hair, and about 5-10% of 3G-“females” have the “male” form of facial and body hair (i.e., Hirsutism, [6,7]) together with the “female” form of breasts.

Regarding brain, behavior, cognition and personality, current data reveal that sex differences in these domains are rarely dimorphic and are often not consistent. Specifically, as pointed out by several authors, for the majority of documented sex differences in the brain of both humans and animals there is considerable overlap between the distributions of the two sexes [8-12]. In fact, in the human brain there is to date no region for which a complete dimorphism has been demonstrated. For example, the intermediate nucleus (InM) of the human hypothalamus (also known as INAH1 and SDN-POA), is on average about twice as large by some measures in males, but there is a tremendous overlap between males and females [13].

I [14] have recently argued that sex differences in brain structure are also not internally consistent (i.e., having one brain characteristic with the “male” form is not a reliable predictor for the form of other brain characteristics). The argument was built on the conclusion, derived from studies in animals, that sexual differentiation progresses independently in different brain tissues, enabling genetically- and environmentally-induced variation in sexual differentiation of different tissues within the same brain (e.g., [15-20]). Indeed, there is ample evidence in the animal literature that environmental factors (e.g., prenatal and postnatal stress [21-32], rearing conditions [33], maternal deprivation [34-37], exposure to drugs [38-40], and anesthesia [41]) can change the form of specific brain characteristics (e.g., size, number of neurons, dendritic morphology, number and size of axons, density of receptors) from the “male” form to the “female” form or vice versa, but that this happens independently or exclusively in select brain regions. For example, Shors and colleagues [31] found a sex difference in the density of apical dendritic spines on pyramidal neurons in the CA1 area of the hippocampus, with dendrites from male rats having fewer spines compared to dendrites from female rats (see Figure 2A, created on the basis of Figure 3 in [31] with pictures obtained from Prof. Tracey J. Shors). Yet, following exposure to 15 minutes of stress, dendrites from stressed males had the “female” form (i.e., high density of spines), whereas dendrites from stressed females had the “male” form (i.e., low density of spines). In contrast, in the basal dendrites of the same neurons there was no sex difference in intact rats, but a sex difference emerged following the 15 minutes of stress, as the latter resulted in increased spine density in males but not in females (see Figure 2B, adopted with permission from [31]).

**Figure 2:**
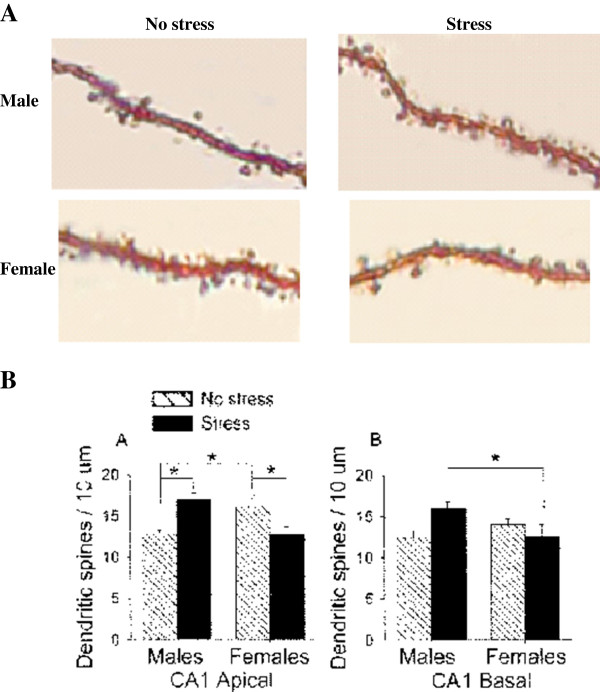
**An example of interaction between sex and environment in determining the structure of brain features. A.** Golgi impregnation of apical dendrites in area CA1 of the hippocampus of male and female rats that did or did not undergo 15 minutes of stress 24 hours before their brains were removed (The pictures were received from Prof. Tracey J. Shors and are from the study reported in Figure 3 in [31]). **B.** The mean and standard error of the mean density of apical and basal dendritic spines on pyramidal cells in area CA1 of the hippocampus of male and female rats that did or did not undergo 15 minutes of stress 24 hours before their brains were removed. Significant differences are marked with asterisks (adopted with permission from Figure 4 in [31]).

Shors et al’s study demonstrates that one should be cautious in the use of the terms “male” form and “female” form when considering brain features, because what is “male” and what is “female” may be different under different environmental conditions. What is “male” and what is “female” may also be different at different stages across the life span (e.g., [11,12]). For example, in the human InM mentioned above, the sex difference disappears entirely after age 45, as the size of the nucleus in older males decreases to match the size of the nucleus in young and older females [13]. It is therefore futile to attempt to determine a “true” “male” form and a “true” “female” form for such brain features (that is, at what age and under which environmental conditions do we observe the “true” “male”/”female” form of such brain features?). Rather, the conclusion from the different lines of evidence cited above is that although the form of many brain features depends on sex, it does not depend only on sex. It is the complex interactions between sex and other factors, such as age and environment, that determine the form of brain features. But even if one maintains a “male” form/”female” form terminology using some type of criterion, Shors et al’s study demonstrates another important conclusion derived from reviewing many such studies, namely, that environmental and experiential events can have different effects on the “male”/”female” form of different brain characteristics, changing some but not others [14]. As a result of these complex interactions of sex and environment, brains are composed of a mosaic of “male” and “female” brain characteristics, rather than being all “male” or all “female” (for example, in the study of Shors and colleagues, pyramidal neurons in CA1 of female rats that were exposed to 15 minutes of stress had apical dendrites with a “male” form and basal dendrites with a “female” form; for a detailed exposition see [14]).

Preceding and in parallel to research on the sexual differentiation of the brain, studies in animals and humans revealed a similar picture with regard to behavior. Thus, already in the 1970’, Goy and Goldfoot [42] noted that there is a considerable overlap in the sexual behavior of males and females in many mammalian species, and Whalen [43] concluded on the basis of his studies in rats that “masculinity and femininity are not unitary processes, but reflect many behavioral dimensions that can be independent” (p. 469). In humans, the conclusion from decades of psychological research is that there are no sex differences on most psychological characteristics (e.g., cognitive abilities, personality traits). Even in the domains in which consistent sex differences are found, there is a considerable overlap in the distributions of 3G-“females” and 3G-“males” (for review see, [44-46]). For example, the effect size (Cohen’s d) of the widely cited sex difference in mental rotation is only 0.47 (i.e., the difference between the average performance of men and women is 0.47 of the standard deviation of this measure, [47]). Moreover, masculine and feminine characteristics (personality traits, attitudes, interests and behaviors that show a sex difference) are not, or are only weakly, correlated (that is, a person who measures high in one domain which shows female superiority may not necessarily measure high on other domains which show female superiority, for review see [48-50]). For example, no correlations were found between subjects’ scores on measures of instrumental traits, expressive traits and sex roles, although there were significant sex differences in these three measures [50]. So also in the domains of behavior, cognition, personality, interests, etc, sex differences are not dimorphic and are not internally consistent. Rather, individuals possess a complicated array of masculine and feminine characteristics, that can not be captured by a uni-dimensional (masculinity-femininity) or a bi-dimensional (masculinity x femininity) model (for review see [48-50]).

It is of interest to note the similar progression of models in the domains of sex and gender and sex and brain (for a review of the history of thought on models of gender see, [51]). Thus, both domains started with uni-dimensional models that were built on the implicit assumption that sex differences are highly consistent, and that therefore they add up to create humans/brains that are distributed along a Masculinity-Femininity or a “male” brain-“female” brain continuum (as depicted for 3G-sex in Figure 1B). In both domains, the uni-dimensional models have been replaced or challenged by bi-dimensional models. In the realm of gender the paradigmatic shift was driven by the finding that subscales of masculinity and femininity were only weakly correlated (e.g., [52,53]). In the study of sexual differentiation of the brain, the view that masculinization and defeminization of the brain are under the sole influence of testosterone was challenged by the discovery, in animals, of entirely distinct and independent cellular processes mediating masculinization and feminization [54]. Finally, in both fields, poor correlations between different features that show sex differences (be they interests, attitudes and cognitive abilities, or the structure of different brain features), has led to the replacement of bi-dimensional models with multi-dimensional or mosaic models. I would like to note, however, that one should not think of the gender mosaic as a direct result of the brain mosaic, because the relations between brain and gender are bidirectional and not unidirectional (e.g., [9]), and because the relations between structure and function in the brain are complex and not straightforward (e.g., [12,55,56]).

A practical suggestion to be taken from this short historical analysis is that studies on the relation between sex and any other domain would greatly benefit from looking not only at sex differences and similarities but also at the correlations, or lack of, between the different features that show a sex difference. In addition, it is strongly recommended to end the indiscriminate use of the term “sexual dimorphism”, which has become synonymous with any average difference between “males” and “females” regardless of the degree of overlap between the two distributions. The term “sexual dimorphism” should be reserved only for brain and gender characteristics that truly show two distinct forms, and in view of the current literature, it seems that when it comes to humans this term should be completely avoided. Last, it may be wiser to use informative terms (such as dense versus sparse, warm versus detached, etc) when referring to the form of brain features or to human characteristics, rather than nominal terms, such as “male” and “female” or masculine and feminine.

In summary, parallel lines of research have led to the conclusion that although there are sex differences in the brain and in behavior, cognition, personality, and other gender characteristics, these sex differences are for the most part not dimorphic and not internally consistent. This is in marked contrast to the almost perfect consistency between the highly dimorphic levels of 3G-sex. Therefore although ~99% of humans are 3G-“males” or 3G-“females”, that is, have all the characteristics of their category, and only ~1% are 3G-“intersex”, when it comes to brain and gender, we all have an intersex brain (i.e., a mosaic of “male” and “female” brain characteristics) and an intersex gender (i.e., an array of masculine and feminine traits).

## Endnote

Most researchers distinguish between the concepts of “sex” and “gender”. The original definition of Unger [57] distinguished between “sex” - the biological differences in genetic composition and reproductive anatomy, and “gender” - the characteristics and traits that are considered appropriate to “males” and “females” (i.e., masculinity and femininity, respectively). The meaning of these concepts has changed in the past three decades in several respects. The definitions of “sex” and “gender” have widened so that “sex” may refer to the biological characteristics of males and females (rather than to genetic-gonadal-genitals characteristics only). Regarding “gender”, it is now recognized that gender is not simply one of the traits people have but rather a social categorization system that affects many aspects of a subject’s life, such as access to power and relations with others (e.g., [58,59]). In parallel, it became clear that “sex” and “gender” are highly intertwined, leading some researchers to use the combined term “sex/gender” (e.g., [60,61]). Here I use the terms “sex” and “gender” as originally defined. However, in order to distinguish my restricted meaning of “sex” from the wider popular meaning, I use the term genetic-gonadal-genitals sex (3G-sex) rather than “sex”. Regarding “gender”, I fully agree with contemporary criticisms of the narrow meaning of the original term, and acknowledge the important role of “gender” as a social categorization system affecting the environment of each individual. However, I relate here only to the masculinity-femininity aspect of “gender” because I focus on the misattribution of meaning to differences between males and females that results from the erroneous assumption that these differences are dimorphic and internally consistent. I do not discuss the source of the differences themselves (e.g., as resulting from the gendered world in which we live).

## Abbreviations

3G-sex: Genetic-gonadal-genitals sex; INAH1: Interstitial nucleus 1 of the human anterior hypothalamus; InM: Intermediate nucleus of the human hypothalamus; SDN-POA: Sexually dimorphic nucleus of the preoptic area.

## Competing interests

The author declares that she has no competing interests.
